# The Christmas Pudding

**Published:** 1887-12-24

**Authors:** 


### The Doctor's Pulpit.

### THE CHRISTMAS PUDDING.

An Englishman likes to feel on every occasion that he is doing right, and he almost always doubts it when he is eating Christmas pudding. It is not, of course, the mere pudding which lies heavily on his conscience, but the turkey and the mince pies and all the other etceteras which go to make up his "Christmas fare." The feasting of the season is a survival from earlier and very different times: times when the majority of our ancestors lived their working lives almost entirely in the open air; when a pound of beefsteak or game pie, washed down with a quart of strong brown ale, would have been thought a moderate and very suitable breakfast with which to begin the day. In those times, not so very remote, the feasting commenced on Christmas Eve, when the yule-log was brought in, and continued not only on Christmas Day but until the new year was fairly entered upon. Even now, in the north of England, a kind of "open house" is kept for all comers, from Christmas Eve until January is well advanced. Geese, turkeys, sirloins, sucking pigs, game pies, mince pies, puddings, cakes, ale, wine, and spirits are abundant in all middle-class houses. Every caller who has the slightest pretence to friendliness, or who does any kind of business for the house, is regaled in the parlour or in the kitchen. "Dull Care" is decidedly driven away, or perhaps the mere approach of the well-remembered season is sufficient to make him take his departure of his own accord, not to return until the second or third week of the new year, when the bills begin to come in.

Is there any reason in all this Christmas feasting? Is it a custom we should establish now if we were at the beginning of our history, instead of being, as we are, well forward, or as some think, hastening to the close of it? Perhaps not. Yet there is something to be said for it; a good deal in fact. Possibly some of our highly ideal and rational contemporaries would like to begin *de novo*, and base all their customs and actions on pure reason. Only we would like to ask—what is pure reason? And whose reason is it to which we are to assign the characteristic of special purity? The moment we begin to apply our abstract principles, that moment our difficulties begin. It is perhaps, on the whole, as well that we have a long, historic past, and possibly it may be better. Even some of our customs which we think unsuited to the present times may have advantages we do not perceive, and without which we might be poorer in many ways than we are.

But is Christmas, with its holidays, its halt in the march, its feastings, its reunions of families and friends, one of those institutions which have outlived their fitness, and which we would willingly see decently buried? We should hardly like to put the question to an assembly of schoolboys, many of whom have daily counted the weeks, the days, and even the hours that lay between them and the day of their departure for the holidays. Their answers might be lacking in that "sweet reasonableness" which is the delight of Mr. Matthew Arnold's soul. Nor would it be kind to ask the wearied City man, or his still more wearied drudge of a clerk, to forego the three days' reprieve from business in his suburban home, or his cold and hurried journey to some far-distant village in north, south, east, or west of this island. We do not, indeed, know where to look for any large class of men who would willingly lose a single hour of the holiday, or a single incident in its history, down to the last mouthful of plum-pudding, or the smallest raisin from the snap-dragon, for any consideration whatsoever. "The Christmas, the whole Christmas, and nothing but the Christmas," would be, from man, woman, and child, the universal cry.

The only persons probably who object to the season and its festivities are those miserable money-grubbers who grudge the few extra shillings or pounds which its many calls will inevitably extract from their pockets; or that equally melancholy and valetudinarian class who have exhausted the potentialities of their gastric energies, and expect nothing from Christmas but overworked stomachs, loaded livers, morose tempers, and dulled brains. It is a question, however, worth some consideration, whether even these are not as much advantaged by the season as their happier-tempered and more robust fellows. Whatever makes the miserly-hearted part with his gold for good objects renders a distinct service to the giver; and even dyspeptics sometimes profit by a compulsory departure from careful dieting, and the indulgence in articles of food which a too rigid self-care has long excluded from their daily menu. Life is not worth living if its rules be too rigidly mechanical. The human creature demands freedom, variety, freshness, self-abandonment, new bodily and mental nutrition, enlarged vision, a more bracing and stimulating physical and intellectual atmosphere. The daily gallop in the park, with the daily corn and hay, may satisfy the horse very well, though even he will find more pleasure in the various herbage and the larger freedom which belong to the summer's run in the well-timbered park or the stretching uplands. But man is something more than a horse; he is endowed with faculties of an essentially different kind; and whatever breaks for him the monotonous and mind-stiffening round of daily business is a circumstance to be regarded with approval, as being serviceable both to body and mind.

Christmas, then, has the double advantage of having pleased and satisfied our ancestors in their more primitive and simpler times, and also of being excellently adapted to ourselves in these days of scientific light, business worry, and life on the principles of the Rule of Three. However we may live by mechanical rules, and however little time we may give to careless and leisurely pursuits, we can never train ourselves to do without periods of easy freedom and holiday festivity like that of Christmas time. We need not try to do without them. If we do, our efforts will only end in permanent injury to ourselves. Every man and woman may not only take his Christmas holiday and join in the prevailing festivities with a good conscience, but also with a rational assurance that he is doing exactly what a reasonable religion and an enlightened interpretation of scientific facts would prompt him to do. "Unbend the bow, throw away the fetters, give rein to innocent natural instincts, lock up business cares and personal anxieties in the jail of forgetfulness, enjoy for a brief period every kind of physical, mental and spiritual good that comes in your way;" this we believe would be the advice of the wisest conclave of divines, physicians, and philosophers which this wise and enlightened nineteenth century could bring together. That being so, the occupant of the Doctor's Pulpit emphasises every word when he wishes all his readers "A Merry Christmas," and a good slice of the pudding when it appears.

